# Application of a multi‐gene next‐generation sequencing panel to a non‐invasive oesophageal cell‐sampling device to diagnose dysplastic Barrett's oesophagus

**DOI:** 10.1002/cjp2.80

**Published:** 2017-08-24

**Authors:** Annalise Katz‐Summercorn, Shubha Anand, Sophie Ingledew, Yuanxue Huang, Thomas Roberts, Nuria Galeano‐Dalmau, Maria O'Donovan, Hongxiang Liu, Rebecca C Fitzgerald

**Affiliations:** ^1^ MRC Cancer Unit, University of Cambridge, Hutchison/MRC Research Centre Box 197, Cambridge Biomedical Campus Cambridge UK; ^2^ Molecular Malignancy Laboratory, Haematology and Oncology Diagnostic Service Addenbrooke's Hospital, Cambridge University Hospitals NHS Foundation Trust Cambridge UK

**Keywords:** Barrett's oesophagus, oesophageal adenocarcinoma, Cytosponge™, dysplasia, hot‐spot panel, biomarker

## Abstract

The early detection and endoscopic treatment of patients with the dysplastic stage of Barrett's oesophagus is a key to preventing progression to oesophageal adenocarcinoma. However, endoscopic surveillance protocols are hampered by the invasiveness of repeat endoscopy, sampling bias, and a subjective histopathological diagnosis of dysplasia. In this case‐control study, we investigated the use of a non‐invasive, pan‐oesophageal cell‐sampling device, the Cytosponge™, coupled with a cancer hot‐spot panel to identify patients with dysplastic Barrett's oesophagus. Formalin‐fixed, paraffin‐embedded (FFPE) Cytosponge™ samples from 31 patients with non‐dysplastic and 28 with dysplastic Barrett's oesophagus with good available clinical annotation were selected for inclusion. Samples were microdissected and amplicon sequencing performed using a panel covering *>*2800 COSMIC hot‐spot mutations in 50 oncogenes and tumour suppressor genes*. S*trict mutation criteria were determined and duplicates were run to confirm any mutations with an allele frequency <12%. When compared with endoscopy and biopsy as the gold standard the panel achieved a 71.4% sensitivity (95% CI 51.3–86.8) and 90.3% (95% CI 74.3–98.0) specificity for diagnosing dysplasia. *TP53* had the highest rate of mutation in 14/28 dysplastic samples (50%). *CDKN2A* was mutated in 6/28 (21.4%), ERBB2 in 3/28 (10.7%), and 5 other genes at lower frequency. The only gene from this panel found to be mutated in the non‐dysplastic cases was *CDKN2A* in 3/31 cases (9.7%) in keeping with its known loss early in the natural history of the disease. Hence, it is possible to apply a multi‐gene cancer hot‐spot panel and next‐generation sequencing to microdissected, FFPE samples collected by the Cytosponge™, in order to distinguish non‐dysplastic from dysplastic Barrett's oesophagus. Further work is required to maximize the panel sensitivity.

## Introduction

Barrett's oesophagus (Barrett's) is the precursor lesion to oesophageal adenocarcinoma (OAC), and it generally progresses to cancer via intermediate stages termed low‐grade dysplasia (LGD), high‐grade dysplasia (HGD), and intramucosal carcinoma (IMC), graded according to the severity of the cellular architecture 1. It is a highly heterogeneous disease with multiple genetically distinct clones identifiable within a Barrett's segment 2. OAC is an aggressive cancer with an extremely poor survival rate with <15% of patients surviving 5 years 3. This is largely attributable to the fact that patients present in the advanced stages of the disease. If Barrett's is diagnosed at the dysplastic stages, it can be treated endoscopically, thus reducing the risk of cancer and improving survival dramatically, even when accounting for lead time bias (>80% 5‐year survival) 4. However, there are challenges with early diagnosis. Firstly, the majority of patients with Barrett's are never diagnosed due to the risk factors being rather common, non‐specific, and reliant on endoscopy. The main risk factors are reflux symptoms, male sex, age > 50, white race, and obesity. The association of OAC with symptoms of gastro‐oesophageal reflux disease (GORD) was first demonstrated in a case‐control study in 1999: odds ratio 7.7 (95% CI 5.3–11.4). Among patients with severe, chronic, recurrent reflux symptoms, the odds ratio was found to be much higher at 43.5 (95% CI 18.3–103.5). More recently, a meta‐analysis has shown that people with GORD are 2.90 (95% CI 1.86–4.54) times more likely to develop Barrett's 5. These results vary significantly, probably because of the way that reflux is defined and categorized, but reflux is clearly a risk factor. Secondly, only 0.33% of patients with Barrett's will progress to OAC 6. This poses the problem of having to survey large numbers of patients who are at very low risk of progression.

Barrett's is usually diagnosed when patients are referred for evaluation of persistent reflux symptoms, or by chance at endoscopy performed for another purpose such as anaemia. Once diagnosed, periodic endoscopic monitoring or surveillance is generally recommended. Surveillance in the United Kingdom currently involves the endoscopic sampling of the Barrett's segment in all four quadrants of the oesophagus every 2 cm with targeted biopsy of visible lesions 7. Whilst this is currently considered the gold standard, there are inherent, unavoidable problems associated with this method. Quadrantic biopsies are unable to sample all of the mucosa, and may potentially miss focal dysplasia, causing a sampling error; endoscopy is invasive, uncomfortable, and time‐consuming with associated high costs. The original Seattle protocol was very important in introducing a systematic approach to surveillance 8. However, more recent studies have shown that 2 cm biopsies may be sufficient 9 and published data show that surveillance protocols are poorly adhered to 10, 11.

To diagnose dysplasia, biopsies are fixed in formalin, processed into paraffin blocks, cut into sections, and stained with haematoxylin and eosin for pathological review. However, diagnosing dysplasia can be subjective 12, 13, 14 and inflammation can mimic the cellular changes observed in dysplasia. There is, therefore, a need to find ways to enhance the current methods used to identify those individuals at increased risk for OAC.

One such alternative to endoscopy for diagnosis and surveillance is the Cytosponge™, a small capsular device that is swallowed and then expands to a sponge in the stomach. As it is removed, by pulling on a string, it collects cells from the whole of the oesophageal epithelium. It samples all the heterogeneous Barrett's clones present 2 thereby removing the sampling bias which occurs with biopsies. Non‐dysplastic Barrett's can be diagnosed using Trefoil Factor Family 3 (TFF3) antibody staining on cells retrieved from the Cytosponge™, which is scored in a binary fashion, thus reducing the difficulties in diagnosing Barrett's using a cytological sample. Studies have shown that the Cytosponge™ is an acceptable, cost‐effective, and relatively accurate method for diagnosing Barrett's with applicability to primary care 15, 16. This could, therefore, solve the first problem of identifying the large proportion of individuals with undiagnosed Barrett's. However, the second problem remains in terms of identifying those at increased risk of cancer. The Cytosponge™ mainly samples surface epithelium and, although cytological atypia may be discerned by an experienced pathologist, a conventional histopathological diagnosis of dysplasia is insufficient alone as a biomarker 16.

Due to the high yield of cells from the Cytosponge™ this could be done as a second tier test for patients diagnosed as TFF3 positive using the same sample. Some data suggest that *TP53* may be a useful biomarker 17, 18, 19, 20, 21; however, the sensitivity (58%) and specificity (85%) of the presence of *TP53* mutation in dysplastic Barrett's Cytosponge™ samples suggests that this single biomarker alone is not suitable for routine clinical use 22. Whilst a panel of biomarkers may achieve a higher sensitivity {e.g. 72% when combined with p53 immunohistochemistry (IHC) 22}, it would be advantageous to use a single platform. Advances in next‐generation sequencing (NGS) techniques mean that instead of focusing on a small number of specific genes, a larger panel may be able to capture the diversity of single nucleotide variants (SNVs) seen in dysplasia and OAC and be useful as a diagnostic tool 20, 23. Previous studies have attempted to find differences between non‐dysplastic and dysplastic Barrett's in order to aid diagnosis but with alternative methods to NGS such as IHC and gene expression profiling 24, 25, 26. Gene expression profiling is not possible due to the formalin‐fixed, paraffin‐embedded (FFPE) preservation of the Cytosponge™ sample which allows for a diagnosis of Barrett's using an immunohistochemical assay for TFF3. Del Portillo *et al* showed that, by using a cancer hot‐spot panel on microdissected FFPE Barrett's biopsy samples, they could differentiate progressors from non‐progressors. Of the 10 patients with sufficient tissue, who progressed to HGD, each had a mutation detected in their HGD biopsy (100%) and 6/8 (75%; 7 had insufficient tissue) had a mutation in their adjacent non‐dysplastic intestinal metaplasia 27. There were no mutations observed in non‐progressors. These observed differences prompted us to consider the potential of coupling this commercial panel with the Cytosponge™ sampling device. It should be noted that microdissection is necessary because the Cytosponge™ samples a large amount of squamous epithelium in addition to any glandular Barrett's epithelium present.

There are certain advantages associated with the use of a hot‐spot panel that make it an appealing potential diagnostic tool for dysplastic Barrett's. The hot‐spot panel used in this study, the Ion AmpliSeq^TM^ Cancer Hotspot panel V2 (ThermoFisher Scientific, Waltham, MA, USA), is already in clinical use and has been fully validated. The panel uses a single pool of 207 primer pairs to perform multiplex PCR covering >2800 mutations in 50 oncogenes and tumour suppressor genes present in the Catalogue of Somatic Mutations in Cancer (COSMIC; http://cancer.sanger.ac.uk/cosmic): *ABL1*, *AKT1, ALK, APC, ATM, BRAF, CDH1, CDKN2A, CSF1R, CTNNB1, EGFR, ERBB2, ERBB4, EZH2, FBXW7, FGFR1, FGFR2, FGFR3, FLT3, GNA11, GNAS, GNAQ, HNF1A, HRAS, JAK2, JAK3, IDH1, IDH2, KDR/VEGFR2, KIT, KRAS, MET, MLH1, MPL, NOTCH1, NPM1, NRAS, PDGFRA, PIK3CA, PTEN, PTPN11, RB1, RET, SMAD4, SMARCB1, SMO, SRC, STK11, TP53,* and *VHL*.

The aim of this pilot case‐control study was to determine whether dysplastic Barrett's cases could be distinguished from non‐dysplastic Barrett's by detecting mutations using the Ion AmpliSeq^TM^ Cancer Hotspot panel V2 on Cytosponge™ samples.

## Methods

### Cohort selection

A retrospective case‐control study design from recent Cytosponge™ trials was chosen to allow for a direct comparison between patients known to have dysplasia and those without. Ethical approval from the East of England‐Cambridge Ethical approval was granted from the East of England‐Cambridge Central Research Ethics Committee (BEST2: Rec. no. 10/H0308/71; Case1:Rec. no. 14/EE/0015). A sample size of 30 in each group was calculated as sufficient for this pilot experiment (a total sample size of 50 would have a power of >90% for detecting the observed difference). Criteria for inclusion were: clear dysplasia status and sufficient remaining tissue (>5 gland groups) which passed sequencing quality control. Barrett's oesophagus of any length was included provided that the case was TFF3 positive (immunohistochemical marker to confirm Barrett's 16) when reviewed by an expert gastrointestinal pathologist (MO'D). Samples were excluded if the patient did not have a surveillance endoscopy with biopsies performed on the same day, with available pathology. For dysplasia status, biopsies from these endoscopies were reviewed in consensus meetings by 2–4 expert pathologists, blinded to the Cytosponge™ result. If a patient had undergone an endoscopy within 6 months prior to the Cytosponge™, and was found to have had a higher grade at that time‐point without subsequent therapy, then this grade was assigned on the assumption that the lesion had not been sampled on the subsequent occasion. Follow‐up data were collected from our own databases and information on progression and mortality collected from Hospital Episode Statistics (up until November 2016).

### Sample preparation and sequencing

All Cytosponge™ samples had been processed into paraffin blocks as described previously 15. The 10 × 4 µm sections of Cytosponge™ tissue were cut on to uncharged slides, with an H&E at each end. Areas of atypia on the H&E were marked by a specialist pathologist (MO'D). Sections were heated to 54°C for 10 min and then deparaffinized in xylene for 5 min. They were dehydrated by emersion in 99% industrial denatured alcohol twice for 3 min each. Samples were microdissected under a microscope by hand using a 21G needle, with the H&E as a guide, and placed in 70% ethanol (supplementary material, Figure S1). Areas of atypia were dissected preferentially, with additional glands taken to give an adequate DNA yield, and the estimated % atypia was recorded. For samples with no atypia, either all glands were dissected, if there were few, or a selection of glands from each quadrant was sampled if Barrett's tissue was prominent throughout the section.

An in‐house, clinically validated protocol was used for DNA extraction. Samples were warmed to 56°C for 10 min to completely evaporate the ethanol. About 7–20 µl of proteinase K digestion buffer [1 ml contains: 100 μl 10× PCR buffer (Applied Biosystems, Waltham, MA, USA; Cat. No. N8080129), 20 µl proteinase K (20 mg/ml; Qiagen, Hilden, Germany; Cat. No. 19131), 0.5 µl Nonidet P 40 (Abbott Molecular, Des Plaines, IL, USA; Ref. No. 30–804808), 880 µl ultrapure water was added to the sample (depending on quantity of tissue), with a drop of mineral oil to prevent evaporation, heated for 5 h at 56°C, and denatured by heating to 96°C for 10 min. Quantification was performed using the Qubit^®^ High Sensitivity assay on the Qubit^®^ 2.0 fluorometer as per the manufacturer's instructions (Invitrogen, Life Technologies, Waltham, MA, USA). A minimum of 5 ng of input DNA was required for sequencing.

Amplicon library preparation was performed using the Ion AmpliSeq™ Library Kit 2.0 (ThermoFisher, Waltham, MA, USA) as per the manufacturer's protocol with target region amplification, amplicon partial digestion with FuPa reagent, barcode adapter ligation, and library purification. Libraries were quantified using the Qubit^®^ 2.0 fluorometer or using Agilent 4200 TapeStation System (Agilent Technologies, Santa Clara, CA, USA). Sequencing was performed on the Ion Torrent PGM platform. Sixteen samples were loaded per chip to give an average of 1000× coverage per amplicon. *TP53* coverage was used for quality control and samples with coverage <100× for each exon were considered to have failed. This reflected the coverage of all mutations and previous studies have considered a coverage of >100× to be adequate 28.

### Mutation analysis

Sequences were aligned to the human hg19 reference genome and mutation calling was performed by the Ion Torrent Suite Version 5.2. Each non‐synonymous variant call was then visually inspected in the BAM file using the Integrated Genome Viewer version 2.3.59. Common single nucleotide polymorphisms (SNPs) in the Single Nucleotide Polymorphism Database (https://www.ncbi.nlm.nih.gov/snp) were excluded from further analysis, if present at either 50% or 100% of the sample, indicating them to have been inherited, as were known false positives caused by non‐specific primer binding 29. Where there was sufficient material, samples were run in duplicate, with repeat library preparation, and SNV calls were made when the mutation was seen in both runs. If cases had an allele frequency (AF) ≥ 12% (see Optimization of mutation calling), > 7 base pairs from the amplicon edge and with no strand bias >3.0 × then these were sufficient to call without performing a duplicate (see data in Results for justification).

### Statistical analysis

Statistical analysis was performed using Graphpad Prism v5 (Graphpad Software Inc., La Jolla, CA, USA). Demographics of the two groups were compared using the Fisher's exact test for sex, unpaired Welch's *t*‐test for age, and Mann–Whitney *U* test for other variables. Correlation was calculated using Spearman's rank. Differences in the mutation rate between different dysplastic groups were compared using the Chi‐squared test. A two‐tailed *P* value of < 0.05 was considered significant. Sensitivity and specificity were calculated in order to consider the ability of the panel to diagnose dysplasia.

## Results

**Figure 1:**
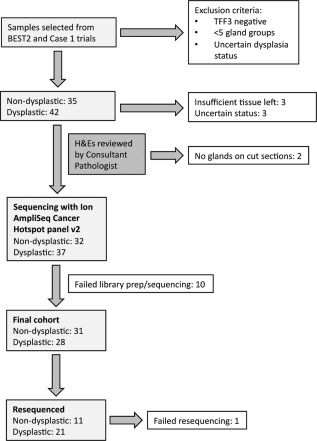
Consort diagram.

Cytosponge™ samples from 31 non‐dysplastic Barrett's oesophagus and 28 dysplastic cases comprising 10 LGD, 6 HGD, 12 IMC samples met inclusion criteria and yielded sequencing data using the Ion AmpliSeq Cancer Hotspot panel v2 on the Ion Torrent platform (Figure 1, Table 1, supplementary material, Table S1).

**Table 1:** Demographics

Cohort demographics	Non‐dysplastic	Dysplastic	*P* value
No. patients	31	28	
Sex – male:female	3.4:1	8.3:1	0.31^c^
Age years – median (range)	64 (16–81)	66.5 (51–81)	0.06^b^
Circumferential length Barrett's (cm) – median (range)	1 (0–10)	4 (0–16)**	0.14^a^
Maximum length Barrett's (cm) – median (range)	4 (1–11)	6 (0–17)**	0.08^a^
Available follow‐up post‐Cytosponge (months) – median (range)	50 (0–63)	41.5 (0–62)	0.11^a^

a Two‐tailed Mann–Whitney test.

Missing data on one patient.

b Two‐tailed unpaired Welsh's *t*‐test.

c Two‐tailed Fisher's exact test.

### Optimization of mutation calling

First, the total numbers of non‐synonymous mutations were determined, as annotated by the software, excluding known non‐pathogenic SNPs. Initially, this revealed mutations in: 13/31 non‐dysplastic Barrett's cases with a combined total of 34 mutations and 24/28 dysplastic cases with a total of 86 mutations. In order to remove mutations introduced by PCR or sequencing error, more common in FFPE tissue, BAM files were interrogated and mutations within 7 base pairs of the amplicon edge (*n* = 6), those with a strand bias >3× (*n* = 7), mutations for which the software had miscalled (*n* = 1), and mutations which were present in multiple samples in the run (*n* = 1; sequencing error) were excluded (supplementary material, Tables S2 and S3). Previous studies using this panel have validated a threshold of 5–10% AF (the frequency of the SNV seen within the sequenced cell population) in fresh cell lines 28 and FFPE tissue 30. These limits were established using the serial dilution of pure cell lines. Singh *et al* 30 only reported a mutation in a cancer at less than 10% AF if it could be validated using another method. In our Cytosponge™ samples, we found that the high sample heterogeneity and lower cellularity meant that it was possible for true mutations to be present at <5% AF, which were impossible to distinguish from false positives. In addition, it has previously been reported that a higher number of false positive mutations can occur with decreased DNA input, poorer quality DNA, and lower read depth 29. This was evident in our samples.

We had 65 mutations below the 5% threshold (after the above exclusion criteria) and therefore 10 samples were run in duplicate, with repeat library preparation, to further define the optimal threshold. Samples were selected to represent the variations in AF and coverage that we were seeing, e.g. samples with a low AF but high coverage and samples with a high AF but low coverage. Two mutations which had been present at 11% (likely owing to low coverage) were not seen in the duplicate. All mutations with an AF ≥ 12% were confirmed on repeat. Below this threshold, it was difficult to ascertain whether a mutation was real or not: some with low AFs < 2% were in the duplicate. Higher coverage did not appear to correlate with the mutation being real (supplementary material, Figure S2). Therefore, we imposed an elevated cut‐off AF of 12% for which we could be confident of calling a mutation rather than potentially include false positives. Duplicates were run for every sample which called mutations with an AF < 12% (supplementary material, Tables S2 and S3). Thus, the final mutation status of each sample was based on the presence of a mutation either ≥ 12%, or in both runs if < 12%. If a duplicate run failed (*n* = 1), or the sample was not repeated because it had at least one mutation ≥ 12% (*n* = 7), then only mutations ≥ 12% were included for that sample. Our intention was to create a binary test: with sample categorized as either mutated (contained at least one mutation) or not mutated.

### Frequency and type of mutations in non‐dysplastic and dysplastic Barrett's

**Figure 2:**
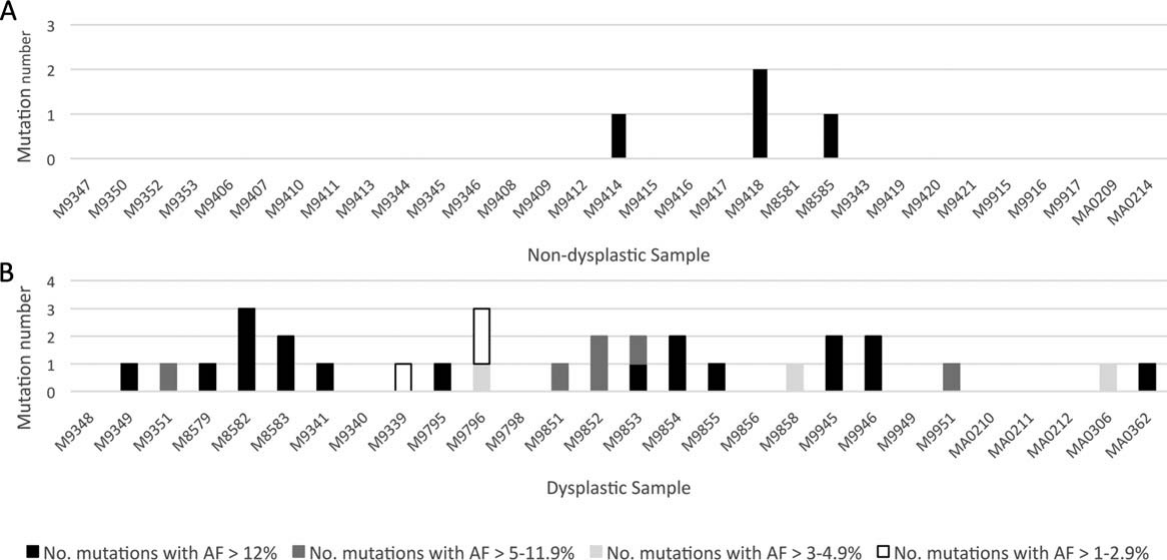
Histograms showing the number of mutations called in each sample (confirmed in duplicate where AF < 12%). (A) Non‐dysplastic Barrett's. (B) Dysplastic Barrett's.

Figure 2 illustrates the mutation profile of the cohort. Using the criteria defined above, 3/31 (9.7%) of non‐dysplastic and 20/28 (71.4%) dysplastic cases were mutated. The median mutation rate per sample in the non‐dysplastic group was 0 (range 0–2), versus 1 (0–3) in the dysplastic group (*p* < 0.0001, two‐tailed Mann–Whitney *U* test).

**Figure 3:**
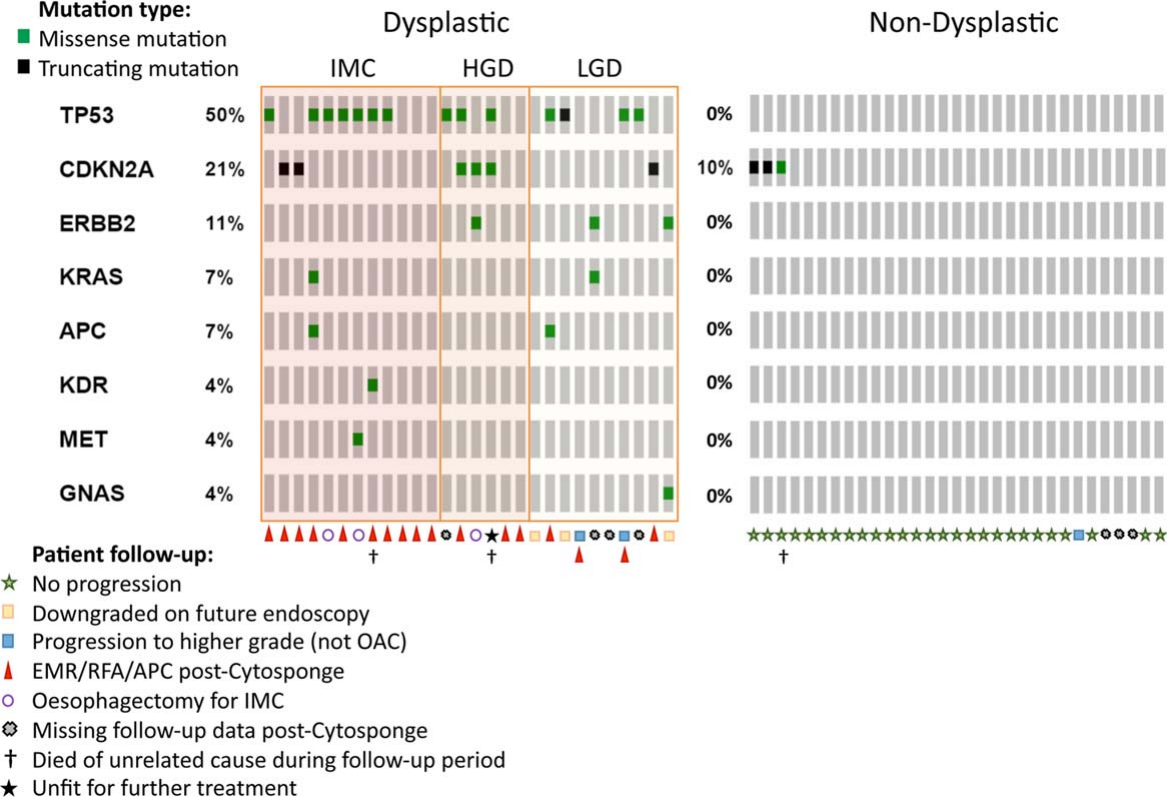
Gene plot showing genes mutated in each sample. Each vertical line of boxes represents one sample.

*TP53* was the most commonly mutated gene, as expected, with 14/28 (50%) of the dysplastic patients harbouring a mutation (Figure 3). *CDKN2A* was mutated at 21.4% and *ERBB2* at 10.7%. Another 5 genes in the panel (*KRAS, APC, KDR, MET, GNAS*) were mutated at a lower frequency. In keeping with our previous data, *SMAD4* was not mutated in any cases in the absence of invasive cancer 21. *CDKN2A* was the only gene mutated in the non‐dysplastic cohort 3/31 (9.7%) in keeping with its known loss early in the natural history of the disease 21.

### Mutation calling is not affected by the presence or percentage of atypia, the grade of the dysplasia or the length of the Barrett's segment

There was a variation in the degree of atypia present in each sample and only 19/28 dysplastic cases had visible atypia on H&E staining. However, there was no significant difference in the total number of mutations per sample in the presence or absence of atypia (*p* = 0.54), nor a correlation between the percentage of atypia present and the total number of mutations called (Spearman's *r* = 0.28; *p* = 0.14). For two of the cases where there was significant atypia, it was possible to compare the calls when sequencing only atypia versus all glands. For case M8582, *KRAS* and *TP53* mutations were identified in both runs but the AF was 1.5–1.7 times higher in the sample microdissected to contain atypia only. One *TP53* mutation, present at 3.4%, was not seen when all glands were taken. This may be because of the lower cellularity, or because it was not present in glands from sections cut further along the paraffin block. Similar results were observed for case M8583 such that the *TP53* mutation AF increased from 24.6 to 46.1% with microdissection. This suggests that, with a genetically heterogeneous disease, microdissection is required to increase the AF sufficiently to make a confident mutation call even when sequencing to high depth.

There was no difference in the number of mutations per sample between the different stages of dysplasia (10 LGD, 6 HGD, 12 IMC; *p* = 0.93) or the maximum length of the Barrett's segment (Spearman's *r* = 0.40, *p* = 0.002).

### Sensitivity and specificity

In order to calculate the sensitivity and specificity of the panel for diagnosing dysplasia, each sample was categorized as being either non‐mutated or mutated. A sample was considered mutated if it had either at least one mutation with an AF > 12%, or a mutation at a lower AF present in duplicate.

The overall sensitivity was 71.4% (95% CI 51.3–86.8) and specificity 90.3% (95% CI 74.3–98.0), positive predictive value 87.0% (95% CI 68.9–95.3%), negative predictive value 77.8% (95% CI 65.8–86.4%). This was recalculated excluding *CDKN2A*, which is known to be mutated in non‐dysplastic Barrett's. Whilst the specificity increased greatly to 100% (95% CI 88.8–100.0%), the sensitivity dropped to 60.7% (95% CI 40.6–78.5%).

### Patient follow‐up

Follow‐up data were available up to 01 November 2016 for 52 of the patients (24 dysplastic, 28 non‐dysplastic). Three patients died of unrelated causes during the period (M9414, M9354, M9946). One of the 28 non‐dysplastic patients (M9420) progressed to LGD in the basal crypts 6 months later but did not have radiofrequency ablation (RFA) and further future biopsies were non‐dysplastic. Seven of the LGD cases had follow‐up data: two of which had no mutations. One of these two cases (M9348) was downgraded to non‐dysplastic on a future endoscopy 6 months later; the other (M9340) progressed to HGD 2.5 years later and was treated with RFA. Of the five patients with mutations, three went on to have RFA (M8583, MA0306), one of whom progressed to HGD (M9858). Two (M9341, MA0362) were downgraded to non‐dysplastic status subsequently.

## Discussion

We have demonstrated here that it is possible to apply a multi‐gene sequencing panel to microdissected FFPE samples collected by the Cytosponge™ with the aim to distinguish non‐dysplastic from dysplastic Barrett's cases. In this pilot case‐control cohort, we achieved a 71.4% sensitivity and 90.3% specificity for diagnosing dysplasia. The sensitivity of this panel is inadequate for use as a clinical test; however, we have shown in principle that a gene mutation panel using NGS can distinguish the two groups and an informed panel is likely to perform better.

The most common mutation in the dysplastic cases was *TP53* in 50% [7/12 (58%) IMC; 3/6 (50%) HGD; 4/10 (40%) LGD]. This rate is lower than that seen previously in the targeted *TP53* sequencing in HGD (72%) 21 but this may be because this panel does not sequence every exon, as discussed below. As shown in previous literature, *CDKN2A* mutation was present in 10% of non‐dysplastic cases 21. We were unable to demonstrate the same *APC* mutation rate found by del Portillo in HGD/OAC biopsies using this panel (2/12; 17%) 27. This is in keeping with whole‐genome and whole‐exome sequencing from the International Cancer Genome Consortium (ICGC) and The Cancer Genome Atlas (TCGA) which did not find *APC* to be significantly mutated in this cancer 20, 31. It should be noted that we have used the histopathological diagnosis as the gold standard to ascertain the sensitivity and specificity of the panel. However, lesions can be misclassified based purely on phenotype and it is possible that the genotype may better predict the long‐term risk of progression to OAC.

These results can be compared to a previous study from our group in which a panel of biomarkers was applied to Cytosponge™ samples in order to stratify patients according to the dysplasia grade (*n* = 468 discovery, *n* = 65 validation). *TP53* mutation was assessed along the entire gene using the Accel‐Amplicon comprehensive panel (Swift Biosciences, NI, USA) followed by sequencing to an average of 10 000 times coverage. *TP53* mutation in combination with p53 IHC 22 had a 72% sensitivity and 83% specificity for diagnosing dysplasia. When the other parameters (atypia, aurora kinase A, clinical parameters) were added they were able to risk stratify patients into three groups. All patients who were classed in the ‘low risk’ group were non‐dysplastic and, in the ‘high risk’ group, the probability of having HGD/IMC was 87%. The AmpliSeq hot‐spot panel used here assesses 492 positions within 7/12 *TP53* exons, covering 1150 COSMIC mutations. It gave a median sequencing depth of 1119 (interquartile range 540–1889) for non‐synonymous mutations and when *TP53* was considered alone gave a sensitivity and specificity of 46.4 and 100%, respectively. The AmpliSeq hot‐spot panel used here informs on the mutation status of multiple genes and has the advantage of requiring only a single platform for use.

The whole‐genome sequencing of 129 OACs by the ICGC demonstrated that most of the genes in this panel are mutated at a low frequency in the cancer (1–16%; *SMAD4* [16%], *CDKN2A* [15%]), compared with the higher rate of *TP53* mutation (81%) 20. Other point mutated genes identified by the ICGC whole‐genome sequencing project which are not in this hot‐spot panel include: *ARID1A* mutated in 17% of samples, *KCNQ3* 12%, and *CYP7B1* 7% 20. Furthermore, whole‐exome sequencing of 72 OACs by TCGA Research Network also identified recurrent mutations in *ARID1A*, *SMAD4*, and *ERBB2* [31]. The whole‐genome and whole‐exome sequencing of Barrett's and cancer pairs have shown the mutational density of SNVs in Barrett's to be 2.8–6.76 SNVs/Mb with mutations occurring in a number of genes which are not in this hot‐spot panel including *EYS*, *SYNE1*, *ARID1A*, *SMARCA4*, *TRIM58* 2, 32. However, the numbers of dysplastic cases in these studies were small. Weaver *et al* used whole‐genome sequencing of OAC to define recurrent mutations and then looked at their frequencies in HGD and non‐dysplastic Barrett's with amplicon sequencing. However, the only gene which defined the boundaries of non‐dysplastic and HGD was *TP53*. Further analysis of the mutation status of the above genes in dysplastic Barrett's may further inform panel design and improve its ability to discriminate between these two stages of pre‐invasive disease. It is also known that the copy number of genes such as *ERBB2* is important in OAC 20 but this cannot be measured accurately using this AmpliSeq panel. Hoogstraat *et al* have developed a custom panel, based on AmpliSeq but with more amplicons covering fewer genes, for which they are able to identify high‐level amplifications at a 25% cell‐line dilution by considering deviations in depth of coverage 33. This might be interesting to explore in the future given the high‐level amplifications seen in this cancer 20, 34, 35.

The Cytosponge™ sample has the advantage over analysing a single biopsy because it takes a sweep from along the entire oesophagus and it has been shown to sample every clone in the Barrett's segment 2. However, the disadvantage of this is that the Barrett's segment is genetically very heterogeneous and most mutations will be present at a low AF. The samples are also formalin‐fixed and paraffin‐embedded. Whilst this is a standard clinical method for sample preparation, the DNA is fragmented and more difficult to sequence and C > T false calls are more likely. In the future, treating FFPE DNA with uracil glycolase to remove these cytosine deamination‐induced changes could minimize this. During the amplification stages of library preparation, there is the potential for introducing mutations, which would similarly be the case if frozen tissue was used. These factors led us to sequence at high depth (1000×) and great care was taken in defining the cut‐off for calling a mutation. Previous studies have found that mutations can accurately be called if they have an AF of 5% or greater 28. The lowest AF mutation which we confirmed was present at 1.7%. However, of the 54 mutations below 5% which were run in duplicate (excluding those which had previously met exclusion criteria) only 6 (11%) were present in the repeat (supplementary material, Tables S2 and S3). This meant that in order to call the low frequency mutations confidently, duplicates were needed, making this impractical and expensive as a clinical test for Barrett's oesophagus. The new methods of library preparation which use molecular barcoding could overcome this problem because they facilitate the accurate calling of SNVs down to an AF of 1%.

Caution is advised for generalizing the results of this study. Samples were chosen carefully for inclusion such that those with fewer than five gland groups or TFF3 negative were excluded and 13.9% (10/72) of samples failed library prep or sequencing (supplementary material, Table S4) so the true sensitivity and specificity could be lower. Using newer samples may possibly overcome some of the difficulties we faced with failure of sequencing in archival samples and microdissecting more sections would increase yield. One of the strengths of this study was that all the techniques and protocols used are currently used in the clinic. However, microdissection is a time‐consuming method of improving cellularity. Whilst it is used clinically for the molecular typing of solid tumours, the areas of interest are usually confined to one area of the tumour so the microdissection is faster. In this study, between 1 and 60% of the whole section were microdissected (median 10%) and so mutations at low allele frequencies would not be seen if the whole section had been extracted.

Overall, while this approach is technically feasible for Cytosponge™ samples, the generic cancer hot‐spot panel alone is unable to diagnose Barrett's dysplasia with a high enough sensitivity to be a useful clinical test. A custom panel in combination with alternative types of marker has the potential to accurately risk stratify Barrett's when used in combination with biopsy or cell collection devices in the future.

## Author contributions statement

AKS, SA, SI: conceived and carried out experiments and analysed data; YH, TR: carried out experiments; NGD: carried out experiments and managed all Cytosponge™ samples; MO'D: reviewed the pathology of all samples; HL, RCF: conceived experiments and analysed data. All authors were involved in writing the paper and had final approval of the submitted and published versions.

## Supporting information

SUPPLEMENTARY MATERIAL ONLINE

**Figure S1.** Cytosponge™ microdissection. ×8 magnification. H&Es are used to guide microdissection of the unstained sections. Areas for dissection are indicated in black with atypical areas marked

**Figure S2.** The difficulty with calling mutations. Sample M9346 had 8 mutations called in the first run of which none of them were seen in the duplicate. The PIK3CA mutation has a high allele frequency (AF) of 11%, and despite the lower coverage of 300, this looked real on inspection of the BAM file in IGV (A). Sample M9796 had all three mutations confirmed in the repeat despite the low AFs. The ERBB2 mutation was 10 base pairs from the edge of the amplicon in a noisy part of the genome as shown in the IGV screen shot (B)

**Table S1.** Microdissection and extraction information for the cohort

**Table S2.** All mutations called for each non‐dysplastic sample and duplicate. Mutations highlighted in grey were used in final calls

**Table S3.** All mutations called for each dysplastic sample and duplicate. Mutations highlighted in grey were used in final calls

**Table S4.** Samples that failed library preparation/sequencing

## References

[1] Schlemper RJ , Riddell RH , Kato Y , *et al* The Vienna classification of gastrointestinal epithelial neoplasia. Gut 2000; 47 **:** 251–255. 10896917 10.1136/gut.47.2.251 PMC1728018

[2] Ross‐Innes CS , Becq J , Warren A , *et al* Whole‐genome sequencing provides new insights into the clonal architecture of Barrett's esophagus and esophageal adenocarcinoma. Nat Genet 2015; 47 **:** 1038–1046. 26192915 10.1038/ng.3357 PMC4556068

[3] Cancer Research UK. *Oesophageal Cancer Statistics*, 2016 Available from: http://www.cancerresearchuk.org/healthprofessional/cancer-statistics/statistics-by-cancer-type/oesophageal-cancer/incidence.

[4] Rice TW , Chen L‐Q , Hofstetter WL , *et al* Worldwide esophageal cancer collaboration: pathologic staging data. Dis Esophagus 2016; 29 **:** 724–733. 27731547 10.1111/dote.12520 PMC5731491

[5] Taylor JB , Rubenstein JH. Meta‐analyses of the effect of symptoms of gastroesophageal reflux on the risk of Barrett's esophagus. Am J Gastroenterol 2010; 105 **:** 1729, 1730–1737, quiz 1738. 20485283 10.1038/ajg.2010.194 PMC2916949

[6] Desai TK , Krishnan K , Samala N , *et al* The incidence of oesophageal adenocarcinoma in non‐dysplastic Barrett's oesophagus: a meta‐analysis. Gut 2012; 61 **:** 970–976. 21997553 10.1136/gutjnl-2011-300730

[7] Fitzgerald RC , di Pietro M , Ragunath K , *et al* British Society of Gastroenterology guidelines on the diagnosis and management of Barrett's oesophagus. Gut 2014; 63 **:** 7–42. 24165758 10.1136/gutjnl-2013-305372

[8] Reid BJ , Blount PL , Feng Z , *et al* Optimizing endoscopic biopsy detection of early cancers in Barrett's high‐grade dysplasia. Am J Gastroenterol 2000; 95 **:** 3089–3096. 11095322 10.1111/j.1572-0241.2000.03182.x

[9] Kariv R , Plesec TP , Goldblum JR , *et al* The Seattle protocol does not more reliably predict the detection of cancer at the time of esophagectomy than a less intensive surveillance protocol. Clin Gastroenterol Hepatol 2009; 7 **:** 653–658, quiz 606. 19264576 10.1016/j.cgh.2008.11.024

[10] Curvers WL , Peters FP , Elzer B , *et al* Quality of Barrett's surveillance in The Netherlands: a standardized review of endoscopy and pathology reports. Eur J Gastroenterol Hepatol 2008; 20 **:** 601–607. 18679060 10.1097/MEG.0b013e3282f8295d

[11] Abrams JA , Kapel RC , Lindberg GM , *et al* Adherence to biopsy guidelines for Barrett's esophagus surveillance in the community setting in the United States. Clin Gastroenterol Hepatol 2009; 7 **:** 736–742, quiz 710. 19268726 10.1016/j.cgh.2008.12.027 PMC3139243

[12] Vennalaganti P , Kanakadandi V , Goldblum JR , *et al* Discordance among pathologists in the United States and Europe in diagnosis of low‐grade dysplasia for patients with Barrett's esophagus. Gastroenterology 2017; 152 **:** 564–570.e4. 27818167 10.1053/j.gastro.2016.10.041

[13] Singh S , Manickam P , Amin AV , *et al* Incidence of esophageal adenocarcinoma in Barrett's esophagus with low‐grade dysplasia: a systematic review and meta‐analysis. Gastrointest Endosc 2014; 79 **:** 897–909.e4, quiz 983.e1–983.e3. 24556051 10.1016/j.gie.2014.01.009

[14] Duits LC , Phoa KN , Curvers WL , *et al* Barrett's oesophagus patients with low‐grade dysplasia can be accurately risk‐stratified after histological review by an expert pathology panel. Gut 2015; 64 **:** 700–706. 25034523 10.1136/gutjnl-2014-307278

[15] Kadri SR , Lao‐Sirieix P , O'Donovan M , *et al* Acceptability and accuracy of a non‐endoscopic screening test for Barrett's oesophagus in primary care: cohort study. BMJ 2010; 341 **:** c4372. 20833740 10.1136/bmj.c4372 PMC2938899

[16] Ross‐Innes CS , Debiram‐Beecham I , O'Donovan M , *et al* Evaluation of a minimally invasive cell sampling device coupled with assessment of trefoil factor 3 expression for diagnosing Barrett's esophagus: a multi‐center case‐control study. PLoS Med 2015; 12 **:** e1001780. 25634542 10.1371/journal.pmed.1001780 PMC4310596

[17] Reid BJ , Prevo LJ , Galipeau PC , *et al* Predictors of progression in Barrett's esophagus II: baseline 17p (p53) loss of heterozygosity identifies a patient subset at increased risk for neoplastic progression. Am J Gastroenterol 2001; 96 **:** 2839–2848. 11693316 10.1111/j.1572-0241.2001.04236.x PMC1808263

[18] Galipeau PC , Li X , Blount PL , *et al* NSAIDs modulate CDKN2A, TP53, and DNA content risk for progression to esophageal adenocarcinoma. PLoS Med 2007; 4 **:** e67. 17326708 10.1371/journal.pmed.0040067 PMC1808095

[19] Davelaar AL , Calpe S , Lau L , *et al* Aberrant TP53 detected by combining immunohistochemistry and DNA‐FISH improves Barrett's esophagus progression prediction: a prospective follow‐up study. Genes Chromosomes Cancer 2015; 54 **:** 82–90. 25284618 10.1002/gcc.22220

[20] Secrier M , Li X , de Silva N , *et al* Mutational signatures in esophageal adenocarcinoma define etiologically distinct subgroups with therapeutic relevance. Nat Genet 2016; 48 **:** 1131–1141. 27595477 10.1038/ng.3659 PMC5957269

[21] Weaver JM , Ross‐Innes CS , Shannon N , *et al* Ordering of mutations in preinvasive disease stages of esophageal carcinogenesis. Nat Genet 2014; 46 **:** 837–843. 24952744 10.1038/ng.3013 PMC4116294

[22] Ross‐Innes CS , Chettouh H , Achilleos A , *et al* Risk stratification of Barrett's oesophagus using a non‐endoscopic sampling method coupled with a biomarker panel: a cohort study. Lancet Gastroenterol Hepatol 2017; 2 **:** 23–31. 28404010 10.1016/S2468-1253(16)30118-2

[23] Dulak AM , Stojanov P , Peng S , *et al* Exome and whole‐genome sequencing of esophageal adenocarcinoma identifies recurrent driver events and mutational complexity. Nat Genet 2013; 45 **:** 478–486. 23525077 10.1038/ng.2591 PMC3678719

[24] Raja S , Finkelstein SD , Baksh FK , *et al* Correlation between dysplasia and mutations of six tumor suppressor genes in Barrett's esophagus. Ann Thorac Surg 2001; 72 **:** 1130–1135. 11603424 10.1016/s0003-4975(01)03005-3

[25] Karamchandani DM , Lehman HL , Ohanessian SE , *et al* Increasing diagnostic accuracy to grade dysplasia in Barrett's esophagus using an immunohistochemical panel for CDX2, p120ctn, c‐Myc and Jagged1. Diagn Pathol 2016; 11 **:** 23. 26926447 10.1186/s13000-016-0473-7 PMC4772649

[26] Varghese S , Newton R , Ross‐Innes CS , *et al* Analysis of dysplasia in patients with Barrett's esophagus based on expression pattern of 90 genes. Gastroenterology 2015; 149 **:** 1511–1518.e5. 26248086 10.1053/j.gastro.2015.07.053

[27] Del Portillo A , Lagana SM , Yao Y , *et al* Evaluation of mutational testing of preneoplastic Barrett's mucosa by next‐generation sequencing of formalin‐fixed, paraffin‐embedded endoscopic samples for detection of concurrent dysplasia and adenocarcinoma in Barrett's esophagus. J Mol Diagn 2015; 17 **:** 412–419. 26068095 10.1016/j.jmoldx.2015.02.006 PMC4484205

[28] Butler KS , Young MYL , Li Z , Elespuru RK , Wood SC. Performance characteristics of the AmpliSeq Cancer Hotspot panel v2 in combination with the Ion Torrent Next Generation Sequencing Personal Genome Machine. Regul Toxicol Pharmacol 2016; 74 **:** 178–186. 26387931 10.1016/j.yrtph.2015.09.011

[29] McCall CM , Mosier S , Thiess M , *et al* False positives in multiplex PCR‐based next‐generation sequencing have unique signatures. J Mol Diagn 2014; 16 **:** 541–549. 25017478 10.1016/j.jmoldx.2014.06.001 PMC4188281

[30] Singh RR , Patel KP , Routbort MJ , *et al* Clinical validation of a next‐generation sequencing screen for mutational hotspots in 46 cancer‐related genes. J Mol Diagn 2013; 15 **:** 607–622. 23810757 10.1016/j.jmoldx.2013.05.003

[31] The Cancer Genome Atlas Research Network . Integrated genomic characterization of oesophageal carcinoma. Nature 2017; 541 **:** 169–175. 28052061 10.1038/nature20805 PMC5651175

[32] Stachler MD , Taylor‐Weiner A , Peng S , *et al* Paired exome analysis of Barrett's esophagus and adenocarcinoma. Nat Genet 2015; 47 **:** 1047–1055. 26192918 10.1038/ng.3343 PMC4552571

[33] Hoogstraat M , Hinrichs JWJ , Besselink NJM , *et al* Simultaneous detection of clinically relevant mutations and amplifications for routine cancer pathology. J Mol Diagn 2015; 17 **:** 10–18. 25445215 10.1016/j.jmoldx.2014.09.004

[34] Gu J , Ajani JA , Hawk ET , *et al* Genome‐wide catalogue of chromosomal aberrations in Barrett's esophagus and esophageal adenocarcinoma: a high‐density single nucleotide polymorphism array analysis. Cancer Prev Res 2010; 3 **:** 1176–1186. 10.1158/1940-6207.CAPR-09-0265 PMC3932797 20651033

[35] Li X , Galipeau PC , Paulson TG , *et al* Temporal and spatial evolution of somatic chromosomal alterations: a case‐cohort study of Barrett's esophagus. Cancer Prev Res 2014; 7 **:** 114–127. 10.1158/1940-6207.CAPR-13-0289 PMC3904552 24253313

