# Supplementary figures and images for: Application of a multi‐gene next‐generation sequencing panel to a non‐invasive oesophageal cell‐sampling device to diagnose dysplastic Barrett's oesophagus
Source: J Pathol Clin Res. 2017 Aug 24;3(4):258–67. doi: 10.1002/cjp2.80 (PMC5653927; doi:10.1002/cjp2.80)

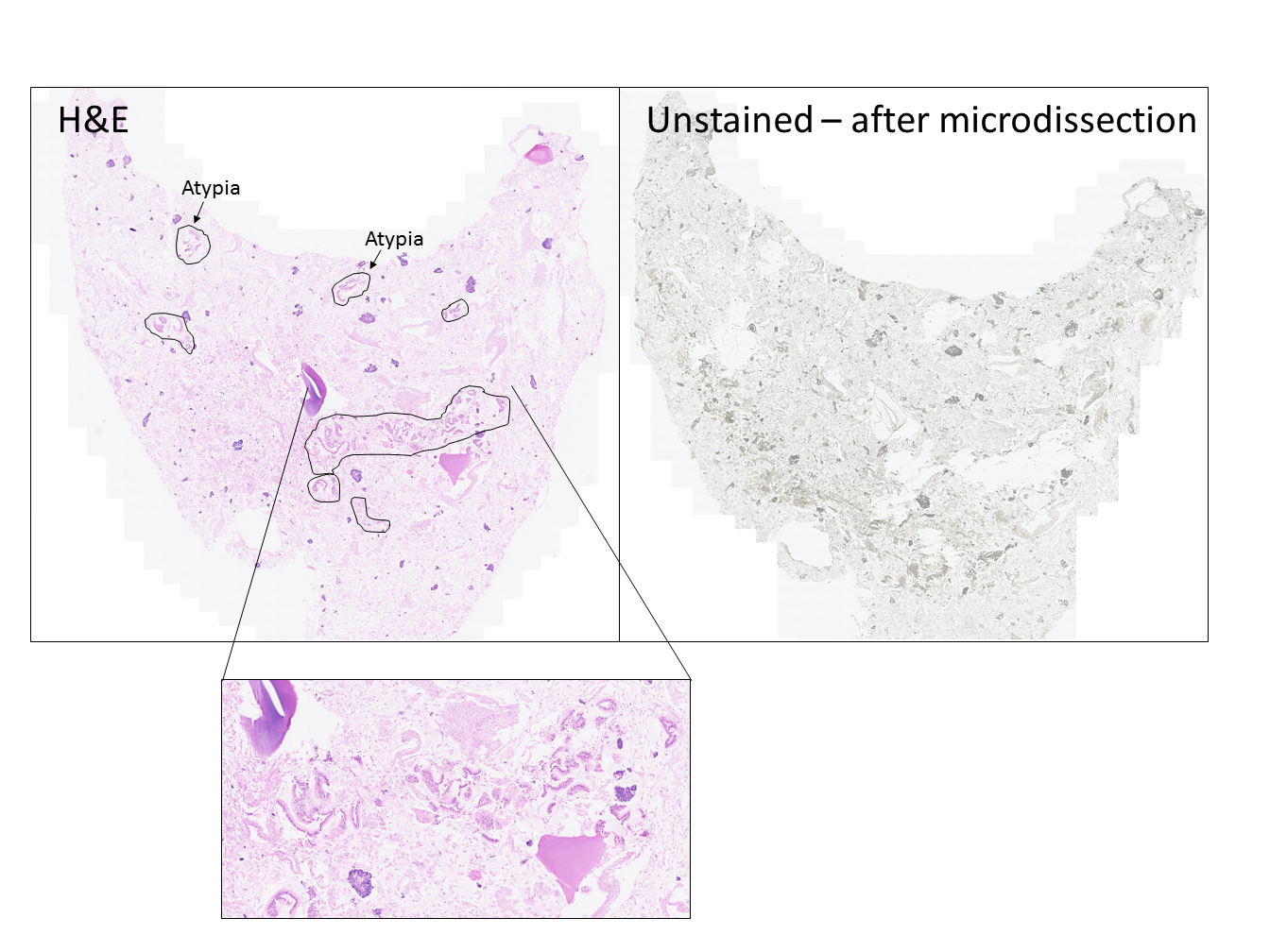

Supplement: Supplementary file 1 — Figure S1. Cytosponge™ microdissection. ×8 magnification. H&Es are used to guide microdissection of the unstained sections. Areas for dissection are indicated in black with atypical areas marked [file CJP2-3-258-s001.tif]

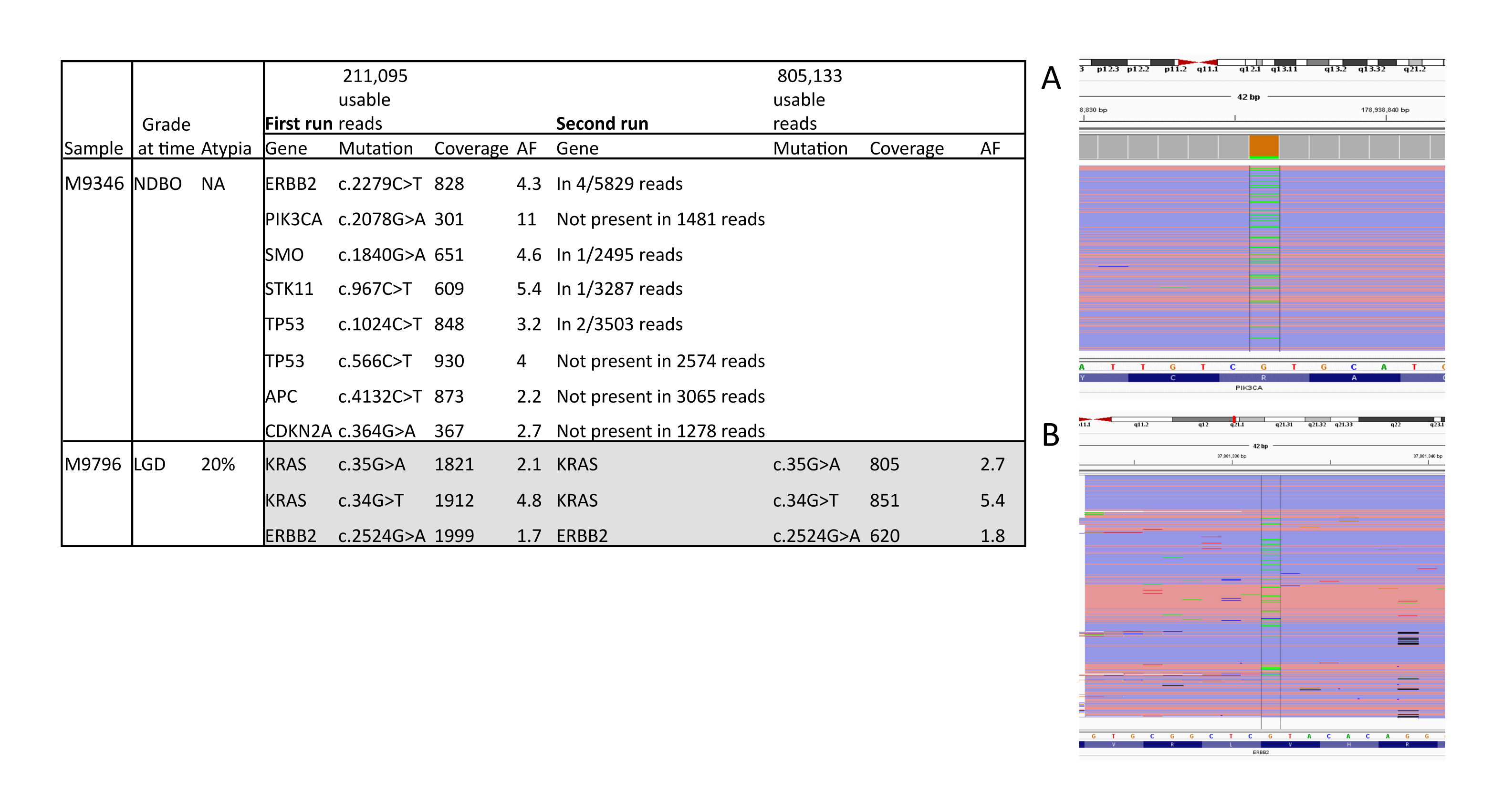

Supplement: Supplementary file 2 — Figure S2. The difficulty with calling mutations. Sample M9346 had 8 mutations called in the first run of which none of them were seen in the duplicate. The PIK3CA mutation has a high allele frequency (AF) of 11%, and despite the lower coverage of 300, this looked real on inspection of the BAM file in IGV (A). Sample M9796 had all three mutations confirmed in the repeat despite the low AFs. The ERBB2 mutation was 10 base pairs from the edge of the amplicon in a noisy part of the genome as shown in the IGV screen shot (B) [file CJP2-3-258-s002.tif]
